# Reflecting on the Spectrum of Involvement: How do we involve patients as partners in education?

**DOI:** 10.1111/medu.15484

**Published:** 2024-08-06

**Authors:** Amber Bennett‐Weston, Simon Gay, Elizabeth S. Anderson

**Affiliations:** ^1^ Stoneygate Centre for Empathic Healthcare, Leicester Medical School University of Leicester, George Davies Centre Leicester UK; ^2^ Leicester Medical School University of Leicester, George Davies Centre Leicester UK

## Abstract

**Background:**

The Spectrum of Involvement describes six levels of active patient involvement in healthcare education. Only at the highest levels are patients described as ‘equal partners’. Although this framework was never intended to be hierarchical, healthcare educators continue to strive towards aspirations for involving patients as ‘equal partners’ in education. However, we do not know what these partnerships mean for all stakeholders and how they can be achieved in practice. This study explores key stakeholders' understandings and experiences of patient partnerships in healthcare education.

**Methods:**

A qualitative case study design was adopted, underpinned by a social constructivist philosophical stance. Semi‐structured interviews were conducted with patients (n = 10) and educators (n = 10) from across a Medical School and a Healthcare School. Five focus groups were held with penultimate year students (n = 20) from across the two Schools. Data were analysed using reflexive thematic analysis.

**Results:**

Three themes were generated: (i) equal partnerships are neither feasible nor desirable; (ii) partnership is about being and feeling valued; and (iii) valuing patients as partners. Patients did not always desire the highest levels of involvement, as ‘equal partners’ in education. All stakeholders agreed that partnership need not be synonymous with equality. Instead, they contended that true partnerships were about valuing patients for their contributions at any level of involvement. Remuneration, student feedback, training and providing institutional access were viewed as important methods of valuing patients as partners.

**Conclusion:**

Patients, educators and students questioned the notion that patient partnerships are only achievable at the highest levels of involvement. Critical application of the Spectrum of Involvement in future research and education is encouraged. This study addresses a gap in the literature, providing tangible approaches to valuing patients as partners that are endorsed by all stakeholders. We propose a model for achieving valued patient partnerships in educational practice.

## Abstract 2

Reflecting on the Spectrum of Involvement, @a_bennettweston @SimonGayGP and @Liz_Anderso describe how to achieve valued patient partnerships in healthcare education.

## 1 INTRODUCTION

Over recent decades, the patient's role in healthcare education has become increasingly active.^1^, ^2^ This follows changes to Western healthcare policy and practice that call for patient‐centred teamworking, integrated care and shared decision‐making.^3^, ^4^, ^5^, ^6^, ^7^, ^8^, ^9^, ^10^, ^11^ Policy changes are steeped in the disability rights movement,^12^ reports of quality and safety failings^13^ and the expectation that medical schools adopt a paradigm of social accountability to the communities they serve.^14^ Whereas patients were once treated as passive learning resources,^15^ they are now expected to be actively engaged as teachers in the training of healthcare professionals.^1^, ^16^, ^17^

Recent reviews have summarised the many roles for patients in healthcare education, which vary greatly in their scope and manner.^2^, ^18^ For example, patients are typically invited to share their lived experiences with students in teaching sessions, but the specific learning objectives and format of those sessions vary greatly.^2^, ^18^ This variation can be at least partly attributed to the practical challenges of patient involvement, including difficulties in securing appropriate funding and a lack of clear guidance on best practice.^2^, ^19^, ^20^, ^21^ Despite variation in patients' roles in education, a plethora of research demonstrates the benefits for enhancing students' empathy, communication skills and supporting the development of patient‐centred professional identities.^22^, ^23^, ^24^, ^25^, ^26^, ^27^

A number of frameworks have been developed to classify and evaluate the degree of patient involvement in healthcare education.^1^, ^28^, ^29^ These frameworks have roots in Sherry Arnstein's^30^ ‘Ladder of Citizen Participation’: seminal work inspired by a political movement for citizens to have greater control over the decisions that affect them. Arnstein proposed progressive levels of involvement in political decision‐making, with aspirations for a redistribution of power between the powerful and powerless to achieve full user control.^30^

In healthcare education, academic debate and discussion on patient involvement has been shaped by the Spectrum of Involvement.^1^ The Spectrum of Involvement^1^ was created based on two earlier frameworks: the Cambridge Framework^28^ and the Ladder of Involvement.^29^ For educators and scholars, the Spectrum of Involvement^1^ has helped to clarify the patient's role in education while encouraging consideration of the contextual attributes that shape interactions between patients, educators and students to support meaningful learning (Table 1). The Spectrum of Involvement^1^ proposes six levels of active patient involvement. Only at the higher levels of involvement are patients described as ‘equal partners’ in healthcare education.^1^

**TABLE 1:** The spectrum of involvement by Towle et al ^1^ including six attributes of involvement (A–F) and six levels of involvement (1–6).

A	B	C	D	E	F
Degree to which the patient is actively involved in the learning encounter	Duration of patient contact	Patient autonomy during the encounter	Training for the patient	Patient involvement in planning the encounter and curriculum	Institutional commitment to involvement
1 Paper‐based or electronic case or scenario: *Patient is focus of a paper‐based, electronic or web‐based case or scenario*	None	N/A	N/A	None	Low
2 Standardised or volunteer patient in a clinical setting: *Patient encounter with student is scripted and serves as an example to reinforce or illustrate learning*	Encounter‐based	None	None	None	Low
3 Patient shares their experience with students within a faculty‐directed curriculum: *Patient is invited to share experience; faculty members plan the encounter but patient determines personal comfort and level of participation*	Encounter‐based	None–low	Brief, simple	None	Low
4 Patient teacher(s) are involved in teaching or evaluating students: *Patient is given preparation for specific teaching role, may actively question students, may be involved in giving feedback and evaluating students' performance*	Variable	Moderate	Structured, extensive	Low–moderate	Low–moderate
5 Patient teacher(s) as equal partners in student education, evaluation and curriculum development: *Patients are involved in many aspects of educational delivery, development and evaluation, beyond specific courses to the curriculum as a whole; this is a true partnership in which patients make meaningful and valued contributions to decision making*	Moderate–extensive	High	Extensive	Moderate–extensive	Moderate
6 Patient(s) involved at the institutional level in addition to sustained involvement as patient‐teachers in education, evaluation, and curriculum development for students: *As (5) above but with additional institutional policies that ensure involvement in decision‐making bodies within undergraduate, graduate, and continuing health professional education*	Extensive	High	Extensive	High	High

Systematic reviews^2^, ^15^, ^18^ have mapped the literature on patient involvement in healthcare education against the Spectrum of Involvement,^1^ demonstrating that the majority of involvement takes place across levels 3–4, where patients share their lived experiences with students. There are exceptions to this, with limited examples of patient involvement at level 5, where patients are involved in the design, delivery and evaluation of healthcare education.^31^, ^32^ These reviews show that patient involvement has benefits for both students and patients.^2^, ^15^, ^18^ For students, patient involvement enhances empathy, communication skills and understanding of patient‐centred care.^2^, ^15^, ^18^ For patients, being involved in healthcare education can provide a sense of fulfilment, improved understanding of their condition and greater confidence in interactions with healthcare professionals.^2^, ^15^, ^18^

Although the Spectrum of Involvement^1^ was not intended to be hierarchical, many educators and scholars continue to frame the higher levels of involvement, and in turn the notion of ‘equal’ patient partnerships, as being aspirational.^2^, ^15^ This finds expression in previous reviews, where the dearth of pedagogic evidence to endorse such partnerships is positioned as a deficit of the literature.^2^, ^15^ A recent theoretical systematic review identified that we lack a strong stakeholder perspective—particularly a strong patient voice—on what these partnerships really mean, whether they are only attainable at the higher levels of involvement and how they can be achieved in practice.^33^ Notably, the Spectrum of Involvement was created without stakeholder input. Moreover, the review^33^ called for the development of a conceptual framework to guide patient partnerships in healthcare education.

This study aimed to understand what it means, to key stakeholders, to involve patients as ‘equal partners’ within healthcare education. To this end, we asked the following question:

How are patient partnerships understood and experienced by patients, educators and students?

## 2 METHODOLOGY

### 2.1 Design

We used a qualitative case study design to acquire an in‐depth understanding of patient partnerships from the perspectives of multiple stakeholders (patients, educators and students).^34^, ^35^ We adopted a social constructivist philosophical stance, whereby meaning is actively constructed, tested and modified by individuals through social interaction.^36^

### 2.2 Setting

The case study comprised one group of patients—‘The Patient and Carer Group’—and their involvement across a Medical School and a Healthcare School at one university in the East Midlands of the United Kingdom. Patients have been involved in healthcare education at the case study site since 1995.^37^, ^38^, ^39^, ^40^ The Patient and Carer Group has 124 members who are involved in various roles including teaching, curriculum development, committee work and admissions. An academic faculty member chairs the group, supported by a patient vice chair and an administrator. A ‘core group’ comprised of patients and faculty members steers the strategy for the wider group. This site was selected for its well‐established patient involvement.^33^

### 2.3 Patient involvement

Given the focus of this study, it was important that patients had a voice in the research process. Accordingly, we set up a reference group comprised of three patients from the Patient and Carer Group who were subsequently excluded from sampling and recruitment. The reference group were involved from the inception of this research to influence the scope of the case study and the content of the topic guides and to ensure that the developing analysis had resonance.^41^, ^42^ Members of this group were not involved in recruitment or data collection; as they were members of the Patient and Carer Group, it was agreed that participants might not be honest about their views of patient involvement in healthcare education if they were to lead the interviews or focus groups.

### 2.4 Sampling and recruitment

Three key stakeholder groups were included in this study: patients, educators and students. Using a purposive sampling strategy, we selected participants for maximum variability on dimensions of relevance to the research.^43^ Patients were recruited to represent a diversity of lived experiences of healthcare and for lengths and types of involvement. A mix of clinical educators and non‐clinical educators (healthcare professionals and scientists) were recruited from each programme (medicine, nursing, midwifery, physiotherapy and operating department practice), along with penultimate‐year students from all Schools.

### 2.5 Data collection

Between November 2021 and May 2022, we conducted individual interviews with patients and educators and focus groups with students. Focus groups were used to explore students' perspectives in core and hidden curricula.^44^ The semi‐structured interviews and focus groups, aided by topic guides, asked questions about involving patients as ‘equal partners’ in healthcare education. In response to the COVID‐19 pandemic, we offered participants the choice between in‐person discussions or online discussions via a video‐conferencing platform. Sampling, data collection and data analysis were iterative and concurrent. We ceased sampling and data collection when we had reached meaning saturation; the point at which we had developed a ‘richly textured understanding’ of the complexities and nuances of the concepts generated through data collection.^45^

### 2.6 Data analysis

The interview and focus group transcripts were uploaded into NVivo 12. One author (ABW) analysed the data using reflexive thematic analysis,^46^ using a blend of inductive and deductive reasoning. We ensured the analysis remained grounded in the data while making connections between participants' accounts and the Spectrum of Involvement.^1^ Data from each stakeholder group were initially analysed separately. Each transcript was coded into meaningful segments of text. These codes were clustered into initial themes for each stakeholder group. Next, overarching themes and subthemes were constructed across the entire dataset. This allowed the data to be synthesised and triangulated as different stakeholder perspectives were layered together.^47^ All authors refined the themes through discussion. Throughout the analysis, we followed guidance for quality in reflexive thematic analysis.^48^

### 2.7 Reflexivity

We met regularly as research team to critically discuss how our subjective contexts, assumptions and values might have influenced the construction of knowledge.^36^, ^49^, ^50^ In particular, we were mindful of having begun this research with a positive view of patient partnerships in healthcare education and sought to avoid projecting this onto participants or their accounts. One author (ABW), who was neither a healthcare educator, student, nor a member of the Patient and Carer Group, conducted all of the interviews and focus groups and led the analysis. Consulting the raw data repeatedly and speaking with the patient reference group ensured that the developing analysis reflected participants' views as opposed to our own.

### 2.8 Ethical approval

Ethical approval for this study was received on 7th October 2021 from the University of Leicester's Medicine and Biological Sciences Research Ethics Committee (reference: 31897‐abw11‐ls:healthsciences).

## 3 RESULTS

There were 40 participants. Ten patients and 10 educators took part in individual interviews (lasting 40–60 min), and 20 students took part in one of five focus group (lasting 30–50 min). Of the 10 patients who participated, six identified as women and four as men. Their ages ranged between 45 and 86 years. Collectively, patient participants represented lived experiences of various cancers, lifelong disabling conditions, learning disabilities, mental health conditions and stroke. All of the patients had been consistently involved in healthcare education at the case study site for at least 3 years; several (n = 3) had been involved for over 10 years. All patients had been involved in small‐group teaching sessions for medical students, nurses and midwives on a range of subjects including communication skills, patient safety and being a carer, and all had been involved in a programme in which one patient is paired with 10 students to engage in online discussions over 10 weeks. Many had also been involved in admissions (n = 6) and were, or had served on, the core group (n = 7). Some patients had been involved in curriculum development or had served on committees for the accreditation of healthcare programmes (n = 3).

Of the 10 educators who participated, four identified as women and six as men. Seven educators had clinical backgrounds, in medicine (n = 3), nursing (n = 1), midwifery (n = 1), physiotherapy (n = 1) and operating department practice (n = 1); three educators had non‐clinical backgrounds as scientists. All of the clinical educators had involved patients in their teaching, and several (n = 3) had worked to develop curricula with patients. None of the non‐clinical educators had directly experienced patient involvement in healthcare education.

Finally, of the 20 students who took part, 15 identified as women and five as men. The students came from medicine (n = 8), nursing (n = 4), midwifery (n = 4) and physiotherapy (n = 4) programmes. All of the students had experienced patient involvement in their teaching and learning. For students on medicine, nursing and midwifery programmes, this typically involved small‐group teaching in which patients would share their lived experience with the opportunity for students to ask questions. For physiotherapy students, patient involvement often involved examining people with real health conditions as opposed to examining simulated patients, who are actors.

Reflexive thematic analysis generated three themes that capture participants' understandings of patient partnerships in healthcare education:

(i) *equal partnerships are neither feasible nor desirable*
(ii) *partnership is about being and feeling valued*
(iii) *valuing patients as partners*

i Equal partnerships are neither feasible nor desirable

Patients, educators and students considered that the majority of their involvement required them to share their lived experiences with students; this reflects levels 3 and 4 on the Spectrum of Involvement.^1^ Based on this, participants argued that there should be greater patient involvement in curriculum development and student assessment but agreed that this should be driven by patients' individual preferences and needs.

… we're certainly involved in some delivery of some subjects … but not very widely across the board I wouldn't say …. (Patient 10)

… so [patients should be involved] in all aspects of curriculum design, delivery, assessment … if the patients themselves want to be involved in those things and it will make a meaningful addition to the student experience. (Non‐clinical educator 1)

… [patients] should be involved in all aspects of our teaching … assessment too …. (Midwifery student 4)

However, participants acknowledged that this would require opening up dedicated space in a tightly packed curriculum and felt that some quality assurance processes would be required to build trust for all stakeholders when patients were involved in assessment.

… everyone's courses are so full, there's a lot of content to get through and you're thinking ‘how would I fit this in?’ (CLINED3)

… I think we could certainly use them [patients] more in the practical components of the OSCEs … but the problem is … obviously controlling and standardising that … across multiple groups of students …. (NONCLINED1)

… no two patients … will be the same and if one of my colleagues had a patient who's presenting very differently to my patient … if we're both being assessed very differently, because patients are very different … I don't think the assessment would be very uniform … I would worry … ‘is this really fair?’ (MEDST1)

Participants rejected the highest levels on the Spectrum of Involvement,^1^ where patients are ‘equal partners’ in education. They interpreted ‘equal’ partnerships as patients and educators having an equal contribution to, influence over and responsibility for, curriculum design, delivery and evaluation. They felt that equality in this sense was neither feasible nor desirable. Without the same qualifications, experience and therefore understanding of healthcare curricula as educators, participants agreed that patients might struggle to contribute as equals.

… when you use the word equal in a context like that it gives the impression that we're of equal status and we're absolutely not. We can't be because of all the things that go with status, such as qualifications, experience …. (Patient 4)

… equal partners … I'm not sure they could be equal … because we've got the wealth of experience of education that they don't …. (Clinical educator 2)

… one patient might have one or two or maybe three conditions, whereas a lecturer will have knowledge of those plus many more … so, if you're putting patients and carers as equal to one lecturer … a patient can't really compare …. (Medical student 1)

Patients recognised that they did not share equal responsibility for healthcare curricula with educators. They understood that it was educators who would be held accountable if healthcare curricula did not address the learning outcomes imposed by governing professional bodies. They understood the cruciality of meeting these outcomes for the quality and safety of healthcare. As such, patients stressed that hierarchy was essential to produce competent healthcare professionals.

… there's a reason I'm the patient and not the tutor … the tutor's role is having the hierarchical call on everything … they take on the responsibility of making sure you all achieve the intended outcomes. Because it's an educational setting, you do have to tick particular boxes and it's people's lives on the line … the way I hold a scalpel is up to me, but for a surgeon to hold a particular scalpel is a bit more important (laughter) … how much does a patient really know? (Patient 6)

Similarly, students expressed that a hierarchy between patients and educators was preferable, whereby an educator would be present in a supervisory role during teaching to keep their learning on track. This view seemed to be strongly linked to students' concerns around patients' competence. Several students suggested that they needed to be prepared for patient involvement, in order to fully appreciate the patient's role in their learning.

… there will be a handful of med students who see that we're in med school to learn about medicine, not the holistic side of it and they won't show and have the same interest and respect for learning from someone who's not from a medical background … I think the reason for having patients involved in teaching needs to be communicated to us at the beginning of our degree by a senior faculty member so that all students really get it. (Medical student 5)

Educators identified inherent academic hierarchies that made achieving true equality impossible. They perceived different levels of power and authority within the faculty and noted that even they were subject to such hierarchies: There would always be someone who had greater influence over decision‐making. As such, educators alluded to the redundancy of striving for ‘equal’ patient partnerships.

… in any meeting … there's still that bit of power and hierarchy … if I had a meeting with my team, as the programme lead we can debate whatever it is all along, but then I'm the one who would have to answer to head of school and head of college, so I'd kind of have that final say …. (Clinical educator 4)

ii Partnership is about being and feeling valued

Having rejected the concept of involving patients as ‘equal partners’ in education, participants considered what partnership truly meant. They argued that partnership need not be synonymous with equality. Instead, partnership was about patients being and feeling valued for their contributions irrespective of the ‘level’ of their involvement.

… I just think in terms of partnership, the work that we do with students just needs to be valued … whatever that may be …. (Patient 1)

Well, it's about being valued. You know I think [partnership is] fairly clear, we value the input of everybody on the course … for the student experience …. (Clinical educator 2)

… if you want people to see patients as partners then … they need to be valued … that has to come from our side [as students] but it also has to come from their side [as educators] too …. (Medical student 5)

Importantly, patients emphasised that by this definition, partnership was possible at any level and should not be reserved exclusively for the highest levels on the Spectrum of Involvement.^1^ They felt this was particularly important given that different patients would desire different levels of involvement depending on their personal circumstances (such as retirement, illness or having established careers) and because different levels of involvement served different purposes.

… in terms of partnerships … I think it would be good to point out to a patient that they're still doing a good job even if they don't want to do everything, because a lot of my colleagues might not want to do curriculum design, or maybe they don't want to be involved in the delivery … it all depends … but that doesn't mean they aren't partners … and different roles contribute different things for the students … one isn't better than the other. (Patient 9)

Participants recognised the value of patient involvement for enhancing students' learning and used this to justify their definition of partnership as being and feeling valued. There was agreement that the value of involvement lay in the authoritativeness of patients' authentic lived experiences, something that educators did not provide. Participants explained that this authenticity enhanced students' empathy and knowledge retention by providing memorable hooks upon which to hang their clinical and biomedical theory and skills.

… if I say something to somebody it's more important than the tutor. From the tutor, a lot will go in one ear and out the other, whereas … if it's a patient talking then the chances are they'll [students will] listen more … it sticks more. (Patient 1)

… I learn so much more from patients than anyone else … I'll speak to a patient and then I'll go home and look up and read about the condition that they've experienced, because straight away I can relate a management or diagnosis to a patient and it makes it so much more memorable …. (Medical student 6)

iii Valuing patients as partners

Participants considered the ways by which they could tangibly value patients as partners in education. Remuneration was viewed as the most important method. Participants explained that remuneration created a sense of equity in patients' relations with educators and students, where there might otherwise be an imbalance.

… one of the first ways of showing value is to be paid for your work … that's quite important … otherwise some people will feel that they're just being used for the information they give … there is that sense of one side, the patient, is giving and the other side, the students, are taking. (Patient 4)

Well, one way [to value patients] is to pay them for their input you know we're getting paid for doing stuff and I think they should be paid too, if they want to …. (Clinical educator 3)

… they deserve some sort of payment. This is beyond a voluntary role … if you want people to see these patients as partners then you can't expect a lecturer who's teaching us on, I don't know, respiratory medicine, to receive a full pay package and then someone to come in and share a massive side to medicine on a voluntary role, that's not OK …. (Medical student 5)

Some patients noted that they were unable to accept payment because it interfered with the receipt of their state benefits. They warned that this could deter new patients from becoming involved and stressed the importance of finding alternative approaches so that all patients felt valued.

… I can't get paid … I got paid once and the whole rigmarole and hassle of my benefits, it just became such a turmoil … if patients do want to be paid, and I imagine a lot of them are on benefits, how would the University be able to assist? A few patients might be deterred just sheerly because … it would affect their benefits … and then how do they feel about being a volunteer? Maybe they could offer grants or something … there's always a way round it …. (Patient 6)

Positive feedback from students and educators was also considered to be important. Participants linked this to communicating the impact of patient involvement, that is, showing patients that students intended to take something from their experiences into practice.

… I think some of the comments that are made by students make us feel valued. It's absolutely fantastic when students say ‘oh I understand what you mean’ … hearing it from the staff is also really brilliant … sometimes it's just about being seen to be doing something of value by the people you perceive to be a step more above you, that's where the real sense of being valued comes from …. (Patient 4)

… impact statements would make patients feel valued I guess … and obviously there will be some recognition from students but also from us, that what they're doing is supporting our students … It's … keeping them informed about the impact of what they'e doing …. (Non‐clinical educator 1)

I think reflecting—a forward reflection—based on what they've told us and how we would use that moving forward. So, we're not just listening but actively listening and showing that their experience matters to us as well …. (Midwifery student 2)

Similarly, the way in which educators spoke to and about patients, particularly in front of students, was considered important for creating valued patient partnerships. Patients recognised that some members of faculty needed help to understand more about teaching with patients as partners.

I think it boils down to the tutor, the way the patient's introduced and what students think they can gain out of it depending on how I've been introduced …. (Patient 6)

I guess it's partly how we talk about them in front of the students … that has an impact on how valued they feel … it's quite easy to make people feel rubbish if you're not careful. (Clinical educator 5)

Patients explained that they did not have the same explicit markers of membership to the university as educators, such as identification badges, email addresses and a physical base. They interpreted this to mean that they were not valued as partners in education and argued that the absence of these markers created practical barriers to their participation as partners.

… partnership … we ought to have our own base and identification badges and emails … I don't think I've ever been in any sort of job where I haven't had a base the same as anyone else in the job that I've worked in (laughter). Remember for some patients this is a job, this is now what they do, they don't do any other work, they do this completely …. (Patient 5)

Finally, participants suggested that providing patients with training might help them to feel like valued partners by supporting their meaningful involvement in healthcare education. Educators emphasised that any training should be co‐created to ensure it met patients' needs.

… being provided with training by the University would be a way of rewarding patients … helping us to develop our skills and ultimately fulfil our role better. (Patient 2).

… patients need to be supported and trained … things like basic teaching skills … but actually ensuring we are asking them what they feel they need to know to be confident in their role …. (Clinical educator 1).

Patients added that having dedicated leadership and administrative support of whom they could ask questions without judgement was essential for facilitating partnership working in practice. They acknowledged, however, that this required financial support to bring into fruition.

… there needs to be levels of support … there needs to be a look at what the overall support needs are of the group in order for us to be partners, and I'm sad to say that's going to involve money and employing people to actually do it …. (PTCR2)

### 3.1 The wheel of patient partnerships

**FIGURE 1:**
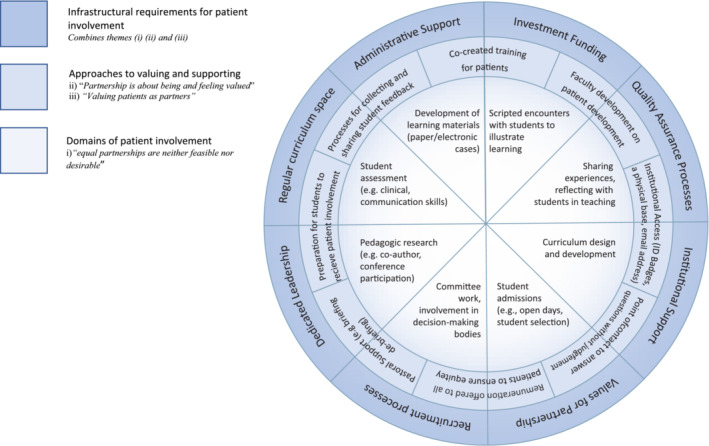
The Wheel of Patient Partnerships. [Color figure can be viewed at wileyonlinelibrary.com]

As the data from each stakeholder group were synthesised separately and then triangulated, the final alignment of the dataset suggests a model for achieving meaningful patient partnerships in healthcare education (Figure 1). We propose ‘The Wheel of Patient Partnerships’ with three layers.

The centre of the wheel summarises the different domains of involvement for patients in healthcare education based on those outlined in the Spectrum of Involvement^1^ and those described by participants in this study under the theme (i) ‘equal partnerships are neither feasible nor desirable’. The second layer encompasses various approaches to valuing and supporting patients as partners before, during and after their involvement, based on the themes on valuing; ‘partnership is about being and feeling valued’ and ‘valuing patients as partners’. Finally, the outer layer captures the infrastructural requirements for valuing and supporting patient involvement, captured across each of the three themes generated in this study.

The Wheel of Patient Partnerships is based on three related assumptions that are grounded in our data. First, this is not a hierarchical or a linear model. All of the components in each of the layers should be available for use, but their selection and implementation should be based on the needs of the patients involved and the desired outcomes of their involvement. Moreover, the components within and between each layer may interact with each other (e.g. administrative support for patient involvement is often contingent on investment and institutional support). Second, there must be at least one component present on each layer of the wheel in any programme of patient involvement. Third and relatedly, it is not necessary for every component on every layer to be present in order to achieve patient partnerships; however, the programme of patient involvement at a healthcare school is likely to be more robust if several components in each layer are present.

## 4 DISCUSSION

We have, for the first time, explored patients', educators' and students' experiences and understandings of partnership and set these in the context of the Spectrum of Involvement.^1^ Our findings challenge well‐established perceptions of patient partnerships and address an important gap in the literature^33^ by illuminating pragmatic approaches to achieving these partnerships in practice. The data suggest a new model to stimulate thinking around, and facilitation and identification of, the different layers and processes needed to support patient partnerships in education.

Involving patients in curriculum development, student assessment and teaching was considered important. This aligns with Spectrum of Involvement's aspirations for involving patients in ‘many aspects of educational delivery, development and evaluation …’.^1^ Importantly, however, participants challenged the concept of ‘equal’ patient partnerships, as outlined in the Spectrum of Involvement.^1^ Instead of being about equality, they contended that true, meaningful partnerships were about valuing patients for their contributions, irrespective of their role. This was considered important because patients make valuable contributions to students' empathy and knowledge retention, a finding that supports previous research.^22^, ^40^ From this perspective, partnership itself is not an ultimate goal but, instead, something that healthcare schools can and should seek to cultivate for all patients involved in education.

Participants suggested ways by which patients could be valued and supported as partners. Remuneration was identified as an important expression of value. However, in line with previous research,^51^ participants noted that alternative remuneration approaches are required for patients in receipt of state benefits to ensure equity. Patients also feel valued when they receive feedback that reflects students' enhanced understanding of the patient perspective. This links to their motivations for becoming involved in healthcare education: to better the next generation of healthcare professionals.^52^, ^53^

Supporting previous research,^54^ we found that training helps patients to feel valued. This highlights the importance of offering training to all patients for partnership working and contrasts the Spectrum of Involvement,^1^ which suggests that training need only be provided for certain types of involvement. Moreover, patients explained that their feeling valued depended on their interactions with educators. Given that previous research^23^ shows that educators require support in working with patients in an educational context, faculty development on patient involvement is required to support the development of patient partnerships.

The Spectrum of Involvement^1^ remains a pragmatic tool to explore and clarify the diversity of roles for patients in healthcare education,^2^ and it continues to inspire and guide those seeking to embark upon patient involvement in healthcare education. However, there are two key aspects of the framework that are challenged by our findings. First, and crucially, is the notion that patient partnerships are only attainable at the higher levels of involvement; in the Spectrum of Involvement,^1^ patients are only described as partners at level five and above. Second, our findings challenge the presentation of partnership as being about equality. Framing patient partnerships in these ways risks reducing the contributions of patients in different roles at ‘lower’ levels of involvement and creates a sense of failure for healthcare schools, where resources are often scarce,^19^ that may not be able to support ‘equal’ involvement in all aspects of education. This is particularly problematic when we consider that the majority of involvement takes place in teaching (which would be considered a mid‐range level of involvement). Our findings suggest that the real failure may lie in neglecting to value patients for their contributions to healthcare education.

Disparity between academic and stakeholder perceptions of patient partnerships might be attributed to the history of the Spectrum of Involvement. We know that frameworks of involvement have roots in Arnstein's ‘Ladder of Citizen Participation’.^30^ This seminal work denotes hierarchical levels of participation and has aspirations for a redistribution of power between the powerful and powerless, such that partnership inherently entails equal power over, and responsibility for, decision‐making. Extrapolated to patient involvement in healthcare education, the conceptualisation of ‘equal’ partnerships seems misplaced, precluding consideration of patients' diverse needs. Not all patients desire the highest levels of involvement and to be ‘equal’ partners; for many, participation itself is the goal.^55^ This is not to say, however, that patients cannot and should not be involved at higher levels, but just that this should not be forced in the interest of achieving so‐called ‘equal’ partnerships and without the appropriate infrastructural support. Although never intended to be hierarchical, the Spectrum of Involvement has, by some educators and scholars, been interpreted as such^2^, ^15^ likely owing to its history. We therefore encourage educators and scholars to reflect on the most appropriate types of involvement for their context and to critically apply the Spectrum of Involvement^1^ in future research and practice.

### 4.1 Implications for research and practice

Educators should not force higher engagement where patients remain content to share their stories in teaching. When designing involvement initiatives, it is therefore crucial that the form of involvement selected aligns with patients' needs and the intended learning outcomes for students, as opposed to being driven by aspirations for achieving the highest level possible on the Spectrum of Involvement.

Healthcare schools should explore equitable approaches to valuing patients as partners in education across all domains of involvement. Our findings suggest that remuneration, feedback from students, providing institutional access (such as identification badges, a physical base and email addresses) are important for valuing patients as partners. Healthcare schools should also consider offering co‐created training to support patients in their roles in education, along with faculty development to support patient–educator interactions that are underpinned by an ethos of partnership. Research is needed to document the development and evaluation of this training to support best practice.

The Wheel of Patient Partnerships (Figure 1) can be used in further research and educational practice. In educational research, the Wheel should be used by authors to clearly identify the domain of patient involvement upon which they are reporting and to be explicit about the ways in which the patients they involved were—or were not—valued and supported, along with the infrastructure required to bring this involvement to fruition. This will contribute to greater consistency in the reporting of patient involvement and will support the sharing of best practice. In educational practice, the wheel is intended to facilitate the consideration of the layers of support and infrastructure required to cultivate and sustain patient partnerships in healthcare curricula. In this way, the wheel provides educators with a ‘menu’ of approaches to patient partnerships that can be selected based on what is appropriate and feasible in their particular contexts.

### 4.2 Limitations

Although conducted rigorously, this study does have limitations. Our case study comprises one model of patient involvement at one UK university, and therefore, the findings may not apply to all healthcare schools. We have, however, provided a rich and situated description of the case study site to enable readers to reflect on the transferability of our findings to their own contexts.^32^

Many participants chose to conduct their interviews or focus groups online due to the COVID‐19 pandemic, although in‐person interviews are considered the gold standard.^56^ Research shows that online interviews with synchronous audio and video are the closest substitute for in‐person interviews in terms of the depth and length of data generated.^57^, ^58^

## 5 CONCLUSION

Patient partnerships in healthcare education have, traditionally, been framed as aspirational, perpetuated by frameworks like the Spectrum of Involvement, which describe patients as ‘equal partners’ only at the highest levels of involvement. Little consideration has been given to what these partnerships mean for all stakeholders, whether they are indeed only attainable at higher levels of involvement and how they can be achieved in practice. Our findings suggest that patient partnerships need not be synonymous with equality and that they can, and should, be achieved at all levels of involvement. We challenge educators and scholars to rethink accepted conceptualisations of patient partnerships in healthcare education and provide a model to support the achievement of these in practice.

## AUTHOR CONTRIBUTIONS

**Amber Bennett‐Weston:** Conceptualization; investigation; writing ‐ original draft; writing ‐ review and editing; methodology; formal analysis. **Simon Gay:** Conceptualization; methodology; writing ‐ review and editing; supervision; investigation. **Elizabeth S. Anderson:** Conceptualization; investigation; methodology; writing ‐ review and editing; supervision.

## CONFLICT OF INTEREST STATEMENT

None to declare.

## ETHICS STATEMENT

Ethical approval for this study was received on 7 October 2021 from the University of Leicester's Medicine and Biological Sciences Research Ethics Committee (reference: 31897‐abw11‐ls:healthsciences).

## Data Availability

The data that support the findings of this study are available on request from the corresponding author. The data are not publicly available due to privacy or ethical restrictions.
